# Assessing the Impact of Nutritional Status on the Quality of Life in Head and Neck Cancer Patients—The Need for Comprehensive Digital Tools

**DOI:** 10.3390/cancers17071128

**Published:** 2025-03-27

**Authors:** Rodica Anghel, Liviu Bîlteanu, Antonia-Ruxandra Folea, Șerban-Andrei Marinescu, Aurelia-Magdalena Pisoschi, Mihai-Florin Alexandrescu, Andreea-Ionela Dumachi, Laurentia-Nicoleta Galeș, Oana Gabriela Trifănescu, Anca-Florina Zgură, Luiza-Georgia Șerbănescu, Cristina Capșa, Andreas Charalambous, Andreea-Iren Șerban

**Affiliations:** 1 Faculty of General Medicine, Carol Davila University of Medicine and Pharmacy, 8 Eroii Sanitari Street, 050474 Bucharest, Romania; rodica.anghel@umfcd.ro (R.A.); antonia-ruxandra.folea@drd.umfcd.ro (A.-R.F.); laurentia.gales@umfcd.ro (L.-N.G.); anca.zgura@umfcd.ro (A.-F.Z.); luiza.serbanescu@umfcd.ro (L.-G.Ș.); 2 Oncological Institute “Alexandru Trestioreanu” Bucharest, 252 Soseaua Fundeni, 022328 Bucharest, Romania; serban.marinescu@yahoo.com (Ș.-A.M.); cristinacapsa@yahoo.com (C.C.); 3 Faculty of Biology, University of Bucharest, 91-95 Splaiul Independentei, 050095 Bucharest, Romania; irensro@yahoo.com; 4 Laboratory for Molecular Nanotechnologies, National Institute for Research and Development in Microtechnologies—IMT Bucharest, 126A, Erou Iancu Nicolae Street, 077190 Voluntari, Romania; mihai.florin.alexandrescu@gmail.com (M.-F.A.); andreeadumachi@gmail.com (A.-I.D.); 5 Department of Preclinical Sciences, Faculty of Veterinary Medicine, University of Agronomic Sciences and Veterinary Medicine, 105 Splaiul Independentei, 050097 Bucharest, Romania; aureliamagdalenapisoschi@yahoo.ro; 6 Department of Automatic Control and Systems Engineering, National University of Science and Technology “Politehnica” Bucharest, 313 Splaiul Independenței, 060042 Bucharest, Romania; 7 Department of Nursing, School of Sciences, Cyprus University of Technology, 15, Vragadinou Str., Limassol 3041, Cyprus; andreas.charalambous@cut.ac.cy

**Keywords:** head and neck cancer, quality of life, nutrition, radiotherapy, digitalization

## Abstract

**Background/Objectives**: Malnutrition is a key determinant of quality of life (QoL) in patients with head and neck cancers (HNCs), influencing treatment outcomes and the occurrence of adverse events (AEs). Despite there being numerous studies on nutritional status and QoL, there is no standardized risk or prognostic model integrating clinical and demographic factors. **Methods:** A literature search was conducted in September 2024 in Scopus, PubMed, and Web of Science, covering studies published between 2013 and 2024. Articles were selected based on their relevance to AEs, nutritional interventions, and QoL assessments in HNC patients. **Results**: The key factors influencing QoL in HNC patients include age, sex, weight, BMI, educational level, and tumor features. Mucositis was identified as the most significant food intake-impairing AE, contributing to malnutrition and reduced QoL. Current QoL assessments rely on descriptive questionnaires, which lack personalization and predictive capabilities. Digital tools, including machine learning models and digital twins, offer potential solutions for risk prediction and personalized nutritional interventions. **Conclusions**: Despite significant research efforts, QoL assessment in HNC patients remains non-uniform, and risk models integrating nutritional status are lacking. A comprehensive, personalized approach is needed, leveraging digital tools to improve nutritional intervention strategies.

## Simple Summary

Patients with head and neck cancer often struggle with eating due to side effects from treatment, which can lead to malnutrition and a lower quality of life. Despite there being many studies on this issue, there is no clear way to predict which patients will be the most affected or how best to support them. The current methods for assessing quality of life are often too general and do not offer personalized insights. Digital tools, including artificial intelligence for predictive models, could help doctors better understand and manage patients’ nutritional needs. By collecting and analyzing more patient data, the medical community will be able to create more effective, individualized support strategies. The findings of this study could lead to better care for cancer patients and provide a framework for future studies on improving nutrition and quality of life.

## 1. Introduction

Head and neck cancers (HNCs) are malignancies found in the oral cavity, paranasal sinuses, nasal cavity, nasopharynx, oropharynx, hypopharynx, salivary glands, and larynx. Notwithstanding the varied locations, head and neck tumors constitute a pretty uniform category for histological categorization. In more than 90% of instances, head and neck cancers are squamous cell carcinomas. Typical risk factors for head and neck squamous cell carcinomas (HNSCCs) include smoking and heavy alcohol use. HNC’s prognostic factors [1,2] are patient-related features (including smoking, alcohol consumption, performance status, and age), and TNM stage, and Human Papillomavirus (HPV) status, especially HPV-16, has been linked to a higher risk of oropharyngeal cancers. HPV-related oropharyngeal cancers differ from those caused by traditional factors, occurring in younger patients without tobacco or alcohol exposure. These cancers have a milder course and fewer distant metastases [3]. As for nasopharyngeal cancers, Epstein–Barr Virus (EBV) infection is a well-known risk factor, with increased levels of circulating EBV DNA being linked to higher mortality and higher distant metastasis rates [4].

According to various reports, in the past decade, head and neck cancers have occupied the sixth or seventh positions on the list of the most common malignancies, although the number of deaths from this cause has decreased by almost 1.5–2 times [5,6,7,8,9]. HNC incidence is higher in less economically developed regions [10], while in Central and Eastern Europe, this group of pathologies has a higher incidence [11]. For example, in Poland, HNC was reported in 2019 as the fourth most common cause of cancer deaths among men [12]. In Romania, a 2023 statistic from the National Institute of Public Health indicates that the incidence of pharyngeal cancer is higher than the European Union average. An estimation from 2020 to 2040 predicts an increase in cancers of the salivary glands, lips, and oral cavity alongside an expected decrease in nasopharyngeal and oropharyngeal cancers among the male population. Concerning mortality, in 2019, oral cavity cancer was the eighth most lethal cancer in Romania [13].

The burden of HNSCC disease is linked to the proximity of tumors to specific anatomical structures, which can result in significant functional impairments due to local invasion. This, in turn, can cause difficulties in speaking, breathing, and eating, thereby diminishing patients’ quality of life (QoL) [14,15,16].

The treatment of HNC patients is inherently interdisciplinary. Radiotherapy (RT), in particular, is an effective therapeutic modality for HNC patients at all clinical stages, whether administered as monotherapy (cT1-2 cN0 cM0) or in advanced stages [17], as it provides enhanced locoregional control [18]. Definitive radiation added to induction and/or concurrent chemotherapy [19] increases the effectiveness of radiotherapy by disrupting the cancer cell’s repair processes and inducing localized lesions of the mucosa [18]. This approach represents a non-surgical therapeutic option for individuals with head and neck cancer [20]. Intensity-modulated radiotherapy (IMRT) is considered the standard of care for the curative treatment of HNSCC patients [21], while helical IMRT has the ability to achieve a more homogenous irradiation of the target volume, offering enhanced sparing of organs at risk and a decrease in reported acute and late toxicities compared to alternative techniques [22,23,24,25,26].

Although combined chemoradiation therapy (CCRT) enhances oncological outcomes, it has not translated into improved nutritional status for HNC patients [27], who endure a wide range of treatment-related toxicities and adverse effects (AEs) alongside chronic comorbidities that require constant monitoring and specialized care [28,29].

Traditionally, the effectiveness of therapy is evaluated by overall survival and/or disease-free survival. However, these are not the only parameters of efficacy; the quality of life during and after therapy is also crucial. This is particularly evident in the ability to mitigate toxicities that lead to adverse effects and comorbidities. For example, when employed in HNSCC treatment, RT may lead to various frequent early or late AEs, such as mucositis, associated [30,31] with difficulties in food intake (chewing and swallowing), xerostomia, dysphagia, dysgeusia, trismus, hoarseness, nausea, vomiting, dermatitis, bone necrosis, soft tissue fibrosis, hearing and speech disorders, fatigue, and pain [32,33,34,35,36,37,38,39,40,41]. Such AEs negatively affect patients’ QoL and may result in treatment cessation [42,43], but most often, these AEs lead to a decrease in the feeding capacity of patients with negative consequences on the nutritional status and, consequently, on the evolution of the disease.

In this review, we explore the impact of nutrition on QoL. Through an exploration of the literature on this topic, we aim to discuss the main factors influencing the occurrence of adverse events (AEs) in patients with head and neck cancers as well as the effectiveness of various interventions in improving QoL by preventing and reducing treatment-related AEs. The main theme is malnutrition as a key component of QoL along with the models used to assess QoL. We argue that it is necessary to have complete and personalized tools obtained through a flexibilization of preventive indicators extracted from current protocols and by building a more extensive dataset about the patient. A more comprehensive approach, which takes into account this wide variety of data and allows for personalized care and prevention, is enabled by digital solutions used in clinical settings whether or not they integrate with the concept of a digital twin.

The concept of a digital twin (DT) has been successfully used in various industrial fields, for example, in the automotive industry [44] to generate synthetic data obtained through high-quality simulations of processes (including wear processes), whose study in laboratory conditions would have been deficient due to a lack of instrumentation or the impossibility of creating experimental conditions close to practical reality. Based on such paradigms, there are also tendencies in medicine to conceptualize physiological and pathological phenomena through complex models (obtained through machine learning), individualized and grouped under the framework of a “digital twin of the patient” [45]. This digital patient should include components dedicated to the goals of targeted and personalized medicine. In the present review, we aim to identify, based on current knowledge, the set of independent data associated with adverse effects related to treatment (output data). The set of inventoried variables could serve to create complex models in different machine learning configurations within studies on cohorts of significant size. Such tested and validated models can be integrated into a digital twin of oncological patients with HNC, serving to personalize treatment, provide precise predictions, and establish truly personalized therapeutic strategies.

In Section 2, Materials and Methods, we briefly present the methodology used to select the articles on which this review is based. In Section 3, Results, we present the main categories related to the presence of AEs in patients with head and neck cancer: sex, age, weight, body mass index (BMI), educational level, etc. In this section, the issue of mucositis as the most important food intake-impairing AE is addressed. Also, within this section, the main methods of QoL evaluation are presented. In Section 4, Discussion, we discuss the set of food intake-impairing and treatment-related AEs and their statistical associations with malnutrition. We also discuss the non-uniformity and gaps of the interventions and the needs for more refined tools for QoL. Approaching these gaps and needs is possible by supplementing new comprehensive digital tools, representing a new era in oncological care.

## 2. Materials and Methods

A comprehensive literature search was conducted in September 2024 in the Scopus, PubMed, and Web of Science databases, encompassing studies published between 2013 and 2024. The search strategy applied the following formula to filter articles and abstracts: (head and neck) AND cancer AND radiotherapy AND quality of life AND nutritional.

**Figure 1:**
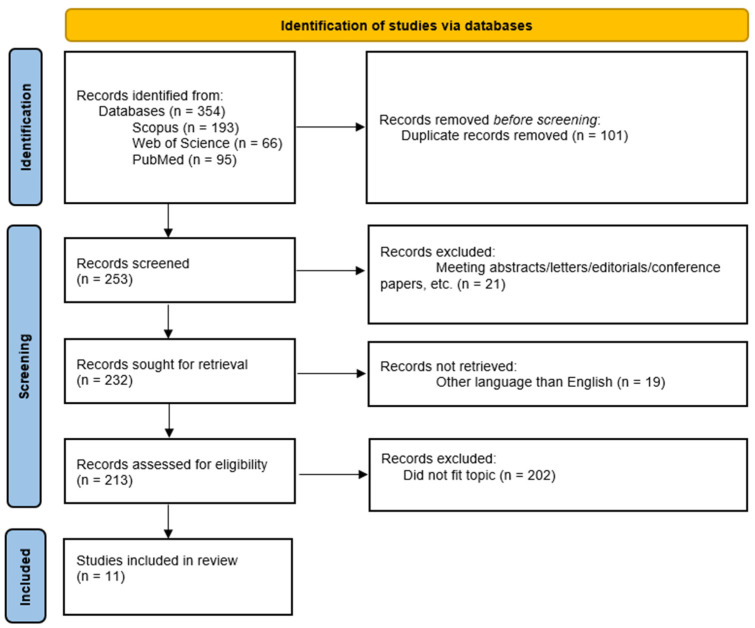
Flow chart showing selection processes.

The study flow chart is presented in Figure 1. Initially, 354 records were identified from the three databases utilized. After removing a total of 101 duplicates and excluding an additional 21 records because they were not reviews or research articles, as well as 19 others because they were written in languages other than English, 213 records were assessed for eligibility. Ultimately, after excluding 201 records that did not fit our review topic, the search yielded 11 research articles and 2 reviews, which have been included in our paper.

In reporting and discussing the most the frequent treatment-related AEs, we used the Common Terminology Criteria for Adverse Events (CTCAE) Version 5.0 [46]. Grades 1–4 of oral mucositis are described within as follows: Grade 1—asymptomatic or mild symptoms, intervention not indicated; Grade 2—moderate pain or ulcer that does not interfere with oral intake, modified diet indicated; Grade 3—severe pain, interfering with oral intake; Grade 4—life-threatening consequences, urgent intervention indicated; and Grade 5—death. Data were extracted by two independent reviewers, with a third intervening as necessary to resolve disagreements. Extracted data included study characteristics (author, year, study design, sample size, independent and dependent variables, and QoL questionnaires employed), population demographics (age, sex, and education level), body mass index (BMI), staging, tumor localization, and treatment characteristics. For uniformity, education level, BMI, tumor localization and staging were reclassified for analysis. Where reclassification was not possible, as was the case for age groups, no further analysis was performed. Chi-square (χ^2^) tests were performed to evaluate associations between observed and expected variables. *p*-values < 0.05 were considered statistically significant.

## 3. Results

### 3.1. Clinical and Demographical Variables Reported in QoL Studies

#### 3.1.1. Sex and Age

The sex and age distributions from the included studies [17,18,29,42,47,48,49,50,51,52] are illustrated in Table 1. A total of 804 patients were included across 10 research articles, with a male-to-female ratio of 76.5% to 23.5% (roughly 3:1). Age, particularly age groups, is inconsistent among the included studies (no two studies have age group categories that can be perfectly superimposed), making it impossible to regroup these patients according to their ages.

#### 3.1.2. Patients’ Education Level

**Figure 2:**
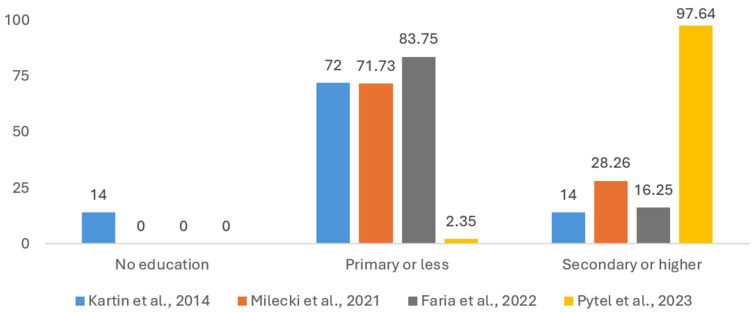
Distribution of patients (%) by three levels of education (no education, primary education or less, and secondary education (high school) or higher education (university), reported in 4 studies: Kartin et al. (2014) [48], Milecki et al. (2021) [42], De Oliveira Faria et al. (2022) [50], and Pytel et al. (2023) [17]).

Education was used as a sociodemographic tool to categorize patients in four studies. No education, primary education or less, and secondary education or higher are the three categories in which the results from these studies were regrouped [17,42,48,50]. As illustrated in Figure 2, most patients have received some form of education, with 55.7% having received primary education or less and 42.02% having received secondary education or higher. In comparison, only 2.28% of patients are non-educated. This could be a factor of bias as the four samples analyzed are statistically different (*p* < 0.001) when compared with each other and are not representative of the general population, with large discrepancies between the two educated groups and the non-educated group.

#### 3.1.3. Weight Loss and Body Mass Index (BMI)

**Figure 3:**
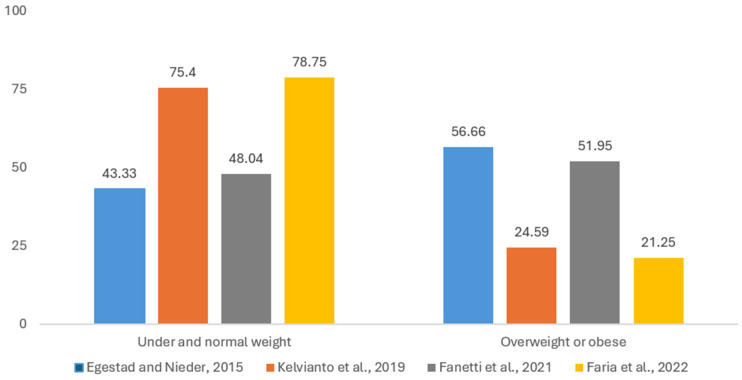
The distribution of patients (%) by body mass index (BMI) according to the results of four studies: Egestad and Nieder (2015) [47], Kelvianto et al. (2018) [18], Fanetti et al. (2021) [29], and De Oliveira Faria et al. (2022) [50]. The patients were divided into 2 main categories: underweight or normal weight (BMI ≤ 24.99) and overweight or obese (BMI ≥ 25).

As BMI categories are not uniformly reported in the included papers, we grouped the patients whose BMI values have been documented into two categories, underweight or normal weight and overweight or obese, in accordance with the World Health Organization’s (WHO) definition of this health-related indicator [53]. Figure 3 is adapted after four studies reporting on BMI [18,29,47,50]. A total of 221 patients are considered underweight or normal weight, while 159 have overweight or obesity. Two of the studies have more patients who are considered underweight and normal weight [18,50] (75.4% and 78.75%, respectively), while the other two [29,47] have a more evenly spread distribution between patients considered under- and normal weight and patients with overweight and obesity. There are statistically significant differences (*p* < 0.001) between the observed and expected distributions in the four groups of patients, potentially due to sampling bias.

#### 3.1.4. Tumor Sites

**Figure 4:**
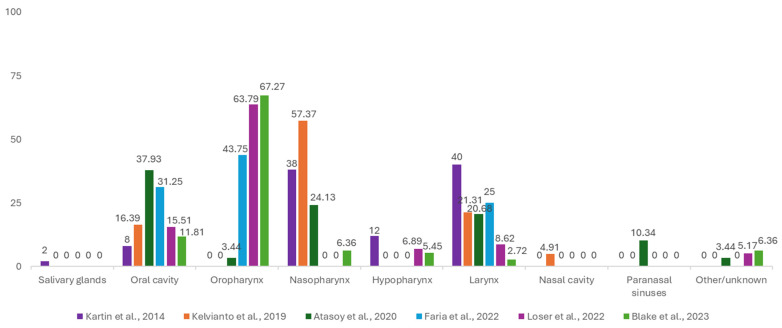
Frequency of head and neck tumors with adverse effects reported in 6 studies: Kartin et al. (2014) [48], Kelvianto et al. (2018) [18], Atasoy et al. (2020) [49], De Oliveira Faria et al. (2022) [50], Löser et al. (2022) [52], and Blake et al. (2023) [51].

Due to the tumor localizations having a non-uniform classification among the original articles that reported on it [18,29,42,47,48,49,50,51,52], they were grouped as follows, as illustrated in Figure 4: oral cavity, oropharynx, hypopharynx, nasopharynx, larynx, salivary glands, nasal cavity, paranasal sinuses, and other/unknown primary sites. For one study [47], the nasopharynx, oropharynx, and hypopharynx sites were grouped into one category, namely ‘pharynx’, and as such, it does not fit our revised classification. Similarly, due to non-uniform reporting, two other studies [29,42] were not included. Special mention also needs to be given to the ‘others/unknown’ category. The choice of name for this subgroup stems from the fact that reporting in the included articles was found to be incomplete in some cases, and this posed difficulties in differentiating between known localizations, reported as ‘other’ due to a small number of cases in the respective patient sample and tumors of unknown primary localization. Salivary glands, nasal cavities, and paranasal sinuses are the three least frequent tumor sites, with the oral cavity, oropharynx, and nasopharynx having the most representation, with more than 70% of all patients having their primary tumors in one of these three sites. Between the six groups of patients, there are statistically significant differences (<0.001), with the observed data not being representative of the general population.

#### 3.1.5. Disease Staging

**Figure 5:**
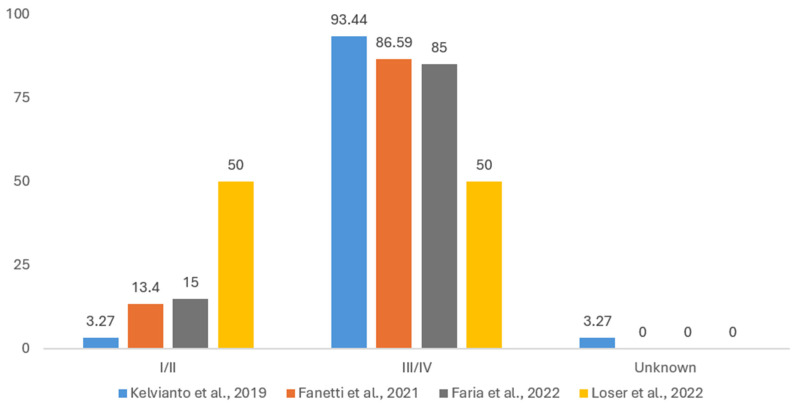
The stages of head and neck tumors according to 4 studies: Kelvianto et al. (2018) [18], Fanetti et al. (2021) [29], De Oliveira Faria et al. (2022) [50], and Löser et al. (2022) [52]. Due to the discrepancies in reporting and the heterogeneity of staging classifications for different localizations of head and neck tumors, groups of stages I–II and stages III–IV were used.

Due to similar non-uniform reporting, we considered two categories for cancer staging, namely stages I/II and stages III/IV, with a third category where staging is unknown. This, however, does not consider that, for the four articles in which cancer staging has been reported [18,29,50,52], different versions of American Joint Committee on Cancer and/or and Union Internationale Contre le Cancer (UICC) staging systems were used. Figure 5 shows that most patients had stages III/IV cancer, while only two patients could not have their disease staged. There are statistically significant differences (*p* < 0.001) between the four groups of patients, likely due to sampling bias.

#### 3.1.6. Treatment Modalities

The four treatment categories considered are RT, chemotherapy, CCRT, and surgery (refer to Table 2). Out of the four categories of treatment reported in the included studies [18,29,47,49,50,51,52], CCRT was the most utilized treatment. For the most part, 60 to 70–71 Gy total RT dose was utilized [29,47,49,51,52], as illustrated in Table 3. As for chemotherapy, three studies [29,51,52] reported on the treatment regimen with doses, while one study [49] only reported that a weekly cisplatin-based regimen was employed without documented dose-related information. Two other studies [18,47] did not report on chemotherapy regimens at all, likely due to chemotherapy-related outcomes not being relevant to their respective analyses.

For the three studies [29,51,52] documenting chemotherapy doses, all depicted cisplatin-based regimens, either a high dose (80–100 mg/m^2^) administered every 3 weeks or a weekly dose of 40 mg/m^2^ of cisplatin. Other chemotherapy regimens depicted include 5-flourouracil and mitomycin C, cetuximab, and TPF (docetaxel-cisplatin-5-fluorouracil) induction followed by weekly cisplatin.

Due to the sometimes sequential nature of HNC treatment, some patients in the analyzed data received more than one type of treatment. As a result, we could not perform comparison tests between the observed values from the included studies.

### 3.2. Oral Mucositis: Impact on QoL Nutritional Aspects, Prevention, Control, and Treatment

Mucositis, whether intrinsic to the disease or arising as an adverse effect of radiotherapy, is the most significant variable influencing the nutrition and QoL axis in patients with HNCs.

**Figure 6:**
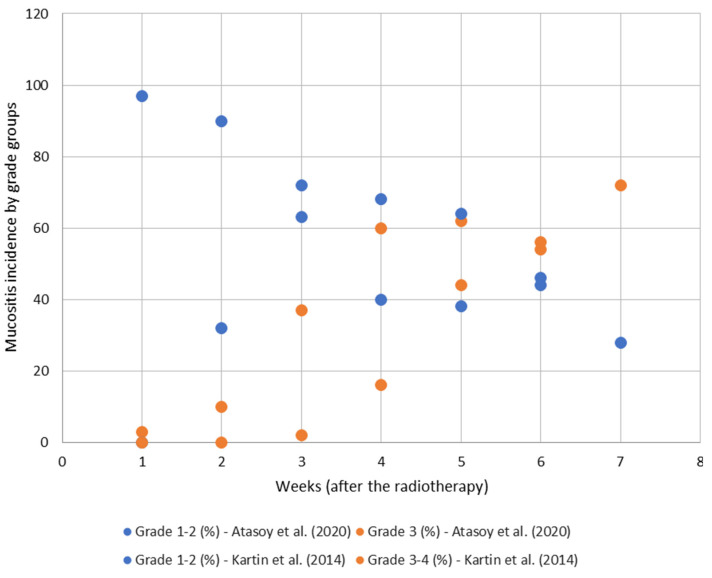
Evolution of cases of oral mucositis Grades 1–2 and Grades 3–4 in post-radiotherapy period as reported by 2 studies [48,49]. Grades 1–2 (non-severe) are presented in blue, and Grades 3–4 (severe) are presented in orange. Results from Atasoy et al. (2020) [49] are represented by circles, and those from Kartin et al. (2014) [48] are represented by squares.

**Figure 7:**
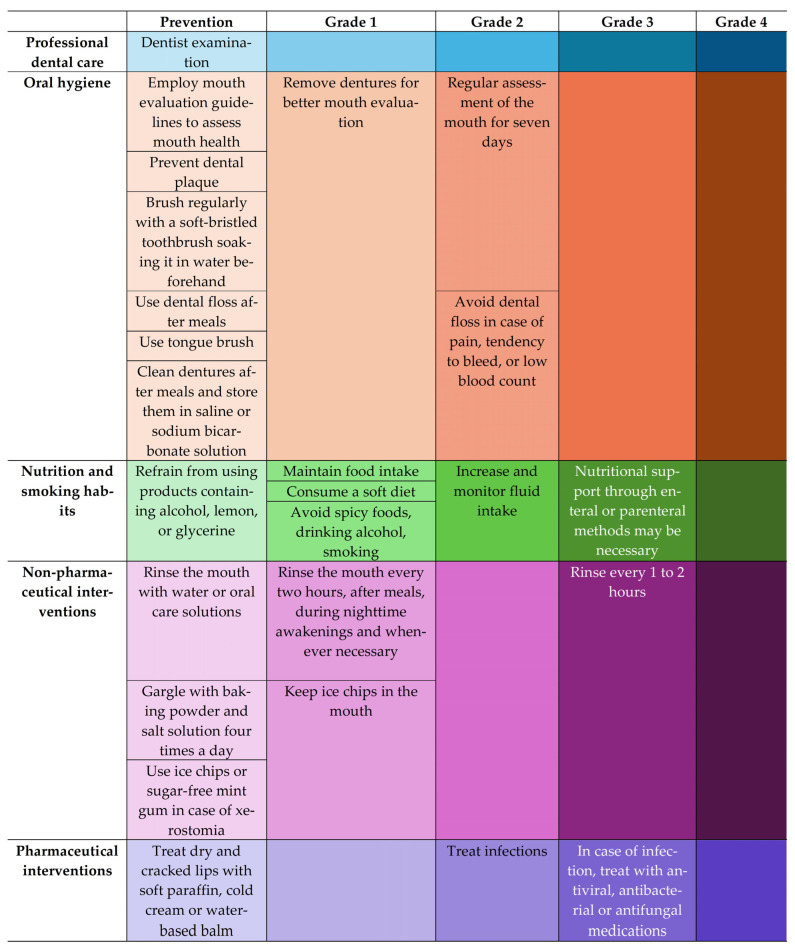
A toolkit of measures for the prevention and control of mucositis in head and neck cancer patients (adapted after [48]). The oral mucositis grading system is defined in the Common Terminology Criteria for Adverse Events (CTCAE) Version 5.0 as follows [46]: Grade 1—asymptomatic or mild symptoms, intervention not indicated; Grade 2—moderate pain or ulcer that does not interfere with oral intake, modified diet indicated; Grade 3—severe pain, interfering with oral intake; Grade 4—life-threatening consequences, urgent intervention indicated; Grade 5—death. We used an increasingly shaded color scale to show the progressiveness of the measures and to emphasize that some higher-grade treatments and control measures (e.g., Grade 3) can be initiated as preventive measures for lower-grade mucositis (in our example, Grade 2) or that control and treatment measures of lower grades can be extended to higher grades.

Figure 6 compares the incidences of mucositis in two studies [48,49] whose objective was to evaluate the quality of life in patients with HNC who were included in our analysis. One of the studies [48] was prospective, while the other [49] was retrospective. The reported results were compared over five weeks during the administration of RT and immediately afterwards. Figure 6 shows that in week 6, the incidence rates of mucositis are comparable, with roughly 45% for Grades 1–2 and approximately 55% for Grades 3–4. However, the two groups had different incidence rates based on severity in week 2 when the reports provided could be compared. Interventions aimed at preventing and treating mucositis (Figure 7) appeared to enhance outcomes, as evidenced by the intratherapeutic follow-up period (weeks 2–5) during which the proportion of cases of non-severe mucositis (Grades 1–2) was greater in patients involved in the interventional study compared to those in the non-interventional study [54,55].

### 3.3. QoL Questionnaires and Associations with Nutritional Status

QoL is usually measured using a questionnaire, which captures both the disease’s impact on a patient’s life and treatment side effects, offering a broader view than standard classifications. Its subjective nature means it reflects only the patient’s perspective. QoL changes are strongly influenced by treatment type and patient adaptability [56,57,58,59,60,61]. The European Organization for Research and Treatment of Cancer questionnaire, EORTC QLQ-C30, supplemented by the H&N-35 module for laryngology patients, is the most validated tool for assessing the QoL of HNC patients [52,62,63,64,65,66,67].

Data from multiple studies [18,42,47,50] reporting on QoL symptoms and parameters using the EORTC QLQ-C30 questionnaire are summarized in Table 4 and Table 5. The data are organized into 15 categories corresponding to individual questions or clusters of questions from the EORTC QLQ-C30. Correlations between QoL parameters and factors such as protein intake or prognostic nutritional index (PNI) were analyzed, and the results are shown in Table 4, while Table 5 presents the median or mean scores at various time points, stratified by age groups, nutritional status, or adherence to intensive nutritional interventions.

Table 6 and Table 7 present data from multiple studies [17,18,42,47,49,50,52], illustrating patient-reported symptoms and QoL parameters from the EORTC QLQ H&N35 questionnaire. For each table, we divided these QoL symptoms/parameters into 20 categories, with most being perfectly superposable onto what each study initially depicted. The outlier from Table 7 that needs mentioning is one retrospective, non-interventional study [49] in which speech-related problems were divided into ‘Talking difficulties’ and ‘Communication’. As we believe that there is virtually no difference between these two categories, we combined them into the more general ‘Speech’ category. For the same study, we integrated taste alteration into the senses category, including smell and taste changes.

BMI—Body Mass Index; RT—Radiotherapy.

The QoL parameters depicted in Table 6 are correlated with factors like protein intake, PNI, HPV status, nutritional assessment tools [Malnutrition Universal Screening Tool (MUST) and Nutritional Risk Screening 2002 (NRS-2002)], and UICC staging, and the ones from Table 7 are reported as the median or mean scores at different time points, based on nutritional statuses, or based on adherence to intensive nutritional interventions.

A notable difference is that none of the included studies reported on all QoL parameters; however, we note that speech, swallowing, and social eating were the three most reported QoL parameters overall, while the least reported were awareness of the disease, followed by sexuality. This discrepancy in reporting, inconsistent reporting metrics (e.g., median vs. mean), and variability in symptom severity reflect differences in patient populations, methodologies, and study designs and are major limitations of the data provided in the table.

## 4. Discussion

### 4.1. Treatment-Related Adverse Effects Leading to Malnutrition

According to our findings in Section 3.2, oral mucositis is a prevalent adverse effect of RT in HNC patients [68], affecting 80–100% [69,70,71] of patients, and it is among the initial consequences of radiation, often presenting two weeks after RT initiation [69,72]. Grades 3 and 4 of mucositis may restrict the capacity to eat, drink, swallow, and talk [73,74], leading to unintentional weight loss and malnutrition [75] in more than 80% of cases [40,76] and associated morbidity in approximately 30% of patients [54,77], with a major impact on medium- and long-term QoL [55,78,79,80].

The cumulative radiation dosage to the oral mucosa is a critical determinant in the development of mucositis. Consequently, a significant side effect is unavoidable with definitive radiation [81]. Cumulative RT dose is a statistically significant predictor for changes in oral mucositis-induced pain scores, while baseline oral pain, concurrent chemoradiation, the volume of oral mucosa, and the mean dose of oral mucosa have been shown to have no statistical significance [49].

Oral mucositis leads to xerostomia, discomfort, burning sensations, infections, and ulcerations. Oral mucositis-induced pain hinders food consumption in patients, negatively impacting nutritional status, which is often characterized by malnutrition [82,83]. Malnutrition most likely induced by mucositis occurs in 40–80% of cancer patients and is a significant contributor to morbidity and death [84,85]. Grades 2 and 3 of mucositis are closely linked to significantly higher enteral nutrition requirements [51]. 

Regular monitoring of mucositis severity is crucial, but oral evaluations are often inadequate [74,86,87,88,89]. Studies suggest that cryotherapy, antiseptic and antifungal agents, topical analgesics, and consistent mouth care may effectively reduce and manage oral mucositis [90,91,92,93], but none of these strategies is firmly recommended [81,94,95,96]. For instance, good oral hygiene, including a soft toothbrush and standardized rinses like sodium bicarbonate and saline, helps reduce oral mucositis in patients [97,98] but is not a standard of care. Due to the clinical and economic impact of oral mucositis, oncology nurses should use evidence-based prevention and treatment practices and educate patients on its management [48].

Another common RT-related AE is xerostomia (the desiccation of the mucous membranes), resulting from impairment of the salivary glands’ secretory activity. Impaired salivation induces pain and complicates eating, leading to malnutrition [99]. It may also result in dental issues and need specialized care [100]. Patients with head and neck cancer may also suffer from dysphagia due to the intricate impairment of the aerodigestive tract’s structure and function [101,102].

Patients with Grade 2 nausea and dysgeusia show a gradual rise in enteral support needs, unlike those with Grade 1, where demand declines by week six, especially for dysgeusia [51]. Grades 1–2 thick saliva lead to a gradual increase in enteral nutrition, tapering off by week six. In contrast, Grade 3 thick saliva results in higher dependence, with 75% of patients requiring substantial support by the end of the observation.

Dysphagia, a severe adverse effect impacting nutrition, shows the largest difference in enteral nutrition needs by toxicity grade [51]. In Grade 3 dysphagia, enteral dependency surges from week 3 of radiotherapy, exceeding 70% by the end of monitoring. Patients with baseline dysphagia require more enteral support than those without, though both show increasing trends. The highest enteral need occurs at the study’s end, with baseline dysphagia patients more likely to remain dependent longer despite there being a gradual decline in use for all. Patients with baseline dysphagia experience a slower decline in enteral nutrition discontinuation, maintaining a significantly higher continuation probability of around 100 days and exhibiting a residual dependency beyond 400 days, whereas those without baseline dysphagia discontinue at a faster rate with minimal continuation after 400 days.

### 4.2. Diagnosis, Evaluation, and Correlates of Malnutrition

In Section 3.1 of this paper, we reported a number of clinical and demographical variables from studies reporting nutrition issues which may impair the QoL of HNC patients. It was observed that such issues affect male patients in most cases; this is obviously related to a higher incidence of HNCs in men, while due to the large variability in the way age is reported, uniform conclusions cannot be drawn about the correlation of age with nutrition. One can imply from Table 1 that there is a threshold age somewhere beyond 40 years old which might indicate a higher malnutrition risk. But this threshold has yet to be obtained from an extensive cohort study. According to the data from the four studies reporting this variable (Figure 3 and Table 2), the level of education of patients with nutrition-related problems is primary education or below.

The prevalence of malnutrition, which is the main consequence of impaired food intake due to treatment-related AEs in HNC patients, varies significantly between studies due to tumor location, treatment severity, and differing standards for identifying malnutrition [103]. Additionally, HNC patients are among the groups who are the most susceptible to malnutrition, not only due to treatment toxicity but also due to dysphagia caused by the tumor itself [6,47,79]. Figure 4 shows that most of the locations of patients included in nutrition studies are located directly on the digestive tract (oral cavity and oropharynx) or in its vicinity (nasopharynx and larynx), which explains why some experience swallowing problems even before the start of treatment. For instance, between 19 and 57% of HNC patients have compromised pretherapeutic nutritional status or malnutrition [16,40,76,104,105,106,107]. Thus, the tumor’s location can heighten the risk of malnutrition as it may cause swallowing or chewing difficulties. A history of alcohol use and/or smoking can further impede oral intake [108,109]. Additionally, factors like the tumor’s metabolic effects or a prolonged history of tobacco and/or alcohol use are also linked to poor nutritional status in these patients [110,111,112,113].

Various other factors can contribute to malnutrition, such as cancer cachexia, which is an inflammation-based syndrome mentioned earlier, inducing proteolysis, lipolysis, and consequent anorexia [15]; increased nutritional demands; psychological influences; and mechanical obstruction due to tumor location or acute multimodal treatment-related toxicities [114,115,116]. Malnutrition is thus more than involuntary weight loss, being described as a subacute or chronic nutritional state where a mix of different levels of under- or over-nutrition and inflammatory processes results in altered body composition and reduced functionality [117]. Between 19% and 57% of HNC patients have compromised pretherapeutic nutritional status or malnutrition [24,51,64,73,74,75,76].

The most direct method, though not the most precise, for quantifying malnutrition is to monitor body weight. A loss of over 5% of a patient’s usual body weight within a month, or 2% within a week, is considered a dependable sign of malnutrition [77]. Patients experiencing weight loss greater than 10% show significantly lower overall survival probabilities compared to those with weight loss of less than 10%, with a markedly sharper decline in the early months following treatment [29].

According to most of the AE studies included in this review, HNC patients reporting nutrition-related QoL impairment were submitted to concomitant chemoradiation (see Table 2). During RT, BMI, along with hemoglobin and albumin levels, had a slight decrease in both normal weight patients and those with overweight [47]. As expected, mucosal changes increase significantly with rising radiation doses regardless of BMI category (Figure 3). At lower doses, a BMI of 25 or higher appears to have a protective effect, as patients experiencing Grades 3–4 mucositis show less severe mucosal changes. [47]. At higher doses, the protective effect diminishes, and the severity of mucosal changes becomes comparable between BMI groups.

Furthermore, a greater risk of weight loss was linked to a Prognostic Nutritional Index (PNI) below 50 and albumin levels below 4.1 g/dl [29], further emphasizing the importance of careful monitoring of nutritional status. The European Society for Clinical Nutrition and Metabolism (ESPEN) states that malnutrition arises from a lack of nutrients, leading to changes in body composition, such as a decrease in fat-free mass (FFM) [52].

Disparities in information sharing have been reported by patients with overweight and obesity who claim to have received less information about what foods and drinks should be avoided over the course of RT compared to their normal or underweight counterparts [47]. This aspect highlights the need for effective communication between healthcare providers and patients.

Although we were able to extract some information on some co-relators of the risk of nutrition-related AEs impacting QoL, it is clear that there is a need to collect more data on HNC patients at different times, namely at the beginning of treatment, during treatment, and at the end of treatment, and when nutritional interventions are involved pre- and post-intervention at different data points. Also, the nature of the data should be diversified by including collected serum variables measuring inflammation, protein, lipid or carbohydrate metabolites, microelements, vitamins, and minerals to which variable indicators of liver and kidney functions should be added. This means a detailed metabolic description of the oncological patient should be created at various moments of time. When this is carried out for an important number of patients, there is a possibility of building multi-variable regressive evolution models or even deep learning models (if the cohorts include hundreds or thousands of patients). Detailed descriptions of patients’ metabolic and nutritional status and associated evolution models enable the creation of a DT, which would open up limitless possibilities for personalized prevention measures.

### 4.3. Relationships of Nutritional Status and Feeding Capacity with QoL

The endpoints of the DT-associated evolutive models could be the variables measured using the questionnaires presented in Section 3.3. Patients at risk for malnutrition can be identified through various screening questionnaires (e.g., NRS-2002, MUST, and Nutriscore) [118,119]. Both NRS-2002 and MUST scores are significant predictors of QoL in multiple domains, like sticky saliva swallowing, weight loss, and the use of a feeding tube [52], emphasizing the importance of nutritional screening and intervention in head and neck cancer patients. Reporting correlations or associations of score variables from these questionnaires by intervention (Table 4 and Table 6) or time of treatment (Table 5 and Table 7) could be achieved when implementing DT using the monitoring metabolic or inflammatory variables.

As mentioned before, a decline in QoL is associated with significant weight loss (≥5%) by the end of RT, and this effect is further exacerbated by the severity of mucositis, which serves as an additional indicator of diminished QoL [49]. Beyond mucositis, an evident cause of nutritional impairment, a weight loss of 5% or more is linked to other descriptors that worsen quality of life, including difficulties in talking or swallowing, altered taste, pain management, communication, social eating challenges, and dry mouth [49]. In the long term, significant weight loss is observed one year after RT, potentially due to factors such as treatment-related side effects, difficulty in eating, or other metabolic changes [42]. Weight loss in advanced cancer stages is associated with poorer QoL [52], an indicator of the need for comprehensive management strategies for late-stage patients. This underscores the risk of deteriorating quality of life due to nutritional issues and, as a result, the necessity for meticulous nutritional monitoring and intervention during and after RT.

The diagnosis of malnutrition necessitates specific criteria: a BMI below 18.5 kg/m^2^, consistent with the WHO’s definition of underweight, or a combination of weight loss with a decreased BMI or diminished gender-specific fat-free mass index (FFMI = FFM/height^2^) [118,120]. Although measuring the BMI is a prevalent anthropometric approach, assessing body composition necessitates bioelectrical impedance analysis, which determines a phase angle that serves to diagnose malnutrition [118,121,122].

When comparing the QoL of HNC patients in relation to their BMI, patients with overweight and obesity generally display comparable QoL scores to those of normal and underweight patients, except for dyspnea, which shows a higher score in individuals with overweight and obesity [47]. Baseline respiratory evaluations by specialized healthcare professionals, particularly in patients with a BMI over 25 who are susceptible to comorbidities affecting the respiratory tract, could be beneficial in detecting these associated conditions, administering necessary treatment in a timely manner and, as a result, improving patients’ quality of life.

Malnourished patients report worse baseline speech, swallowing, sticky saliva, and social eating scores when compared to their well-nourished counterparts. The appetite loss score is higher for well-nourished patients at the end of treatment [52]. These findings underscore the need for early nutritional intervention to mitigate the impact of malnutrition on treatment outcomes and QoL. At baseline, swallowing difficulties and coughing are worse in patients adherent to intensive nutritional care, though this finding might mean patients may have maintained adherence as a result of these baseline symptoms [50].

RT is a treatment modality with a particular impact on clinical variables that more or less affect nutritional status and feeding capacity. Physical, role, and cognitive functions are worse 1 year after RT as compared to before RT, while fatigue, loss of appetite, and constipation also register worsening scores post-RT [42]. Physical function improves as the PNI increases, while dyspnea also enhances with a higher PNI [18]. Total protein intake is negatively correlated with a loss of appetite, fatigue, and nausea and vomiting, emphasizing the importance of adequate protein intake for improving quality of life. This positive correlation with the PNI highlights the significance of this index, which takes into account both nutritional and immune status parameters.

Also, in relation to RT, QoL feeding capacity-related variables such as pain, speech and swallowing difficulties, and social eating are worse at the end of treatment as opposed to at the beginning [49]. Higher scores are reported for sense alteration, mouth opening, xerostomia, and weight loss one year after RT as opposed to before RT initiation, while the use of painkillers decreases one year post-RT [42]. Similarly, speech, swallowing, sense of taste, coughing, and malaise are worse after RT than before, while the use of pain medications decreases after RT, weight loss is more severe after RT, and social eating and contact improve post-treatment [17]. This highlights the possible negative effects of RT on certain symptoms associated with QoL and the necessity for AE prevention during RT to maintain or enhance QoL in the long term.

### 4.4. Prevention and Interventions—Non-Uniformity and Gaps

Ensuring proper nutrition prior to and during treatment can reduce weight loss, prevent dehydration, and improve tolerance, thereby minimizing treatment interruptions [32,77,123]. As per the ESPEN guidelines for nutrition in cancer patients, BMI, nutritional intake, and weight changes should be evaluated as early as the confirmed cancer diagnosis and then repeated on a case-by-case basis as needed [124].

Inadequate nutrition due to decreased calorie and protein intake further aggravates existing nutritional and therapeutic challenges [14] and weakens the immune system’s capacity to operate efficiently [125,126], thus heightening the likelihood of infections, hospital admissions [127], and treatment delays, which may lead to unfavorable treatment results [16,114,128] such as worse prognosis, decreased treatment tolerance with treatment interruptions [127], reduced physical functioning [129], deterioration of QoL [130,131,132,133], more severe (Grades 3/4) late RT-induced toxicities [134], and poorer disease-specific survival [104,131,135].

If oral intake through counseling or oral nutrient supplements does not improve, the ESPEN’s guidelines for nutrition in cancer patients recommend more invasive feeding methods like enteral nutrition, and if that, too, proves to be inadequate, parenteral nutrition is recommended [124]. An increase in the need for enteral nutrition support has been linked to three types of chemotherapy, namely high-dose cisplatin, weekly cisplatin, and cetuximab. High-dose cisplatin has been associated with a slightly higher proportion of patients requiring enteral nutrition by the end of the monitoring period, and although cetuximab and weekly cisplatin show comparable trajectories, they remain slightly lower than high-dose cisplatin [51].

Nutritional counseling, which includes symptom management and dietary advice, is a key first-line intervention for cancer patients [114,136]. Personalized intensive dietary care involving oral nutritional supplements, offering energy and protein, may support patients unable to meet nutritional needs through diet alone. Corticosteroids and progestins to increase appetite or the administration of long-chain N-3 fatty acids to stabilize body weight and improve food intake have been recommended for patients [114]. Research suggests that supplements can boost intake [137], but their impact on outcomes like mortality, treatment tolerance, and quality of life remains unclear [103,114,138]. Some national-level designed guidelines provide detailed recommendations for managing nutrition in head and neck cancer patients [139], emphasizing QoL and treatment tolerance as critical goals. However, evidence to support their guidance on oral nutritional interventions was limited during development, which is not surprising since previous reviews found data to be insufficient or inconsistent evidence from randomized controlled trials limiting definitive conclusions [103,140].

In HNSCC patients undergoing CCRT, nutritional counseling throughout treatment and for up to three months post-treatment proved effective in preserving or even slightly improving anthropometric parameters, such as the skeletal muscle index and FFMI, despite their initial decline during therapy [141]. This post-treatment recovery underscores the crucial role of continued nutritional interventions, not only during treatment but also in the follow-up phase, to support recovery and improve body composition.

Beyond the need for trials to establish the effectiveness of nutritional interventions, we argue that there is a need for unified and uniformly applied assessment instruments for the impact of nutritional status on QoL and survival.

Beyond the type of nutritional intervention, it is also necessary to establish the timing of the intervention. The enteral nutrition timing (prophylactic vs. reactive) and delivery method (gastrostomy vs. nasogastric tubes) failed to resolve weight loss in HNSCC patients, showing that significant weight loss persists regardless of the approach used [142,143], but even with proactive gastrostomy tube placement before treatment, nutritional outcomes did not improve, and adherence to prescribed enteral nutrition was low [144,145]. This situation is caused mostly by the absence of uniform criteria for initiating enteral nutrition support. Some guidelines suggest starting support when oral intake falls below 60% of energy needs [114,139]; however, studies rarely adhere consistently to these criteria. Initiation varies widely from as little as 1 kg of weight loss [146] to waiting until a 5 kg weight loss or less than 50% of energy needs is met.

Weight loss is often used as a clinical trigger for starting enteral nutrition support, reflecting prolonged inadequate oral intake, but it may not always be the best measure for prompting nutrition intervention. Preventive and nutritional support measures should be initiated much earlier, and their intensity should be personalized according to a series of factors that remain to be defined in interventional studies [147]. Patients with enteral nutrition experience feelings of confusion, fear, and anxiety while also recognizing its critical role during treatment. For some, weight loss is a source of shame or embarrassment, while others, particularly those previously overweight, view it as a positive outcome [148]. Psychological factors should not be excluded from the preliminary assessment of the need to initiate invasive nutritional interventions, and these should be preceded by emotional, psychological, and spiritual support interventions.

Swallowing problems, pain, xerostomia, as well as social eating improve with adequate total protein intake [18], showing that sufficient protein in the patients’ diet could benefit their QoL. Moreover, better mouth opening is correlated with a higher PNI [18], signifying the importance of proper monitoring of albumin and leucocyte levels, which are used to calculate the PNI. These findings highlight that early nutrition interventions could prove useful in improving QoL.

Lower scores related to teeth problems and the need for food supplements are documented for patients over the age of 60 [42]. Professional consultations with a dentist or nutritionist, along with regular oral health assessments, could significantly enhance QoL for patients under the age of 60.

### 4.5. Bettering QoL Evalution in HNC Patients by Digital Solutions

#### 4.5.1. Digitalization in Healthcare

A tool that has gained significance in recent years for aiding decision-making and patient monitoring is digital twin (DT) technology. This technology creates a virtual representation of the physical world, integrating components such as cloud storage, task automation, artificial intelligence models, and decision-making capabilities [149]. The use of real-time physiological data, medical history, and individual health profiles through DT technology could assist healthcare professionals in identifying pattern discrepancies and early AEs and promote proactive interventions for better patient outcomes [150,151]. The utilization of DT technologies, particularly the incorporation of non-medical factors like social, emotional, and environmental assessments besides the medical records and medical history of the patient could prove important in drafting personalized care plans for patients and contribute to QoL improvement [152,153]. Of course, this implies not only a systematic data collection but also a change in healthcare personnel training. Recent advances have been made (i.e., TRANSiTION project, https://www.europeancancer.org/eu-projects/resource/transition) to train healthcare professionals for better use of the digital tools dedicated to patient management.

#### 4.5.2. Nutrition Status and Feeding Capacity in Current QoL Evaluations

QoL reflects patients’ views, in the context of the disease, on physical health, mental state, autonomy, and social ties [154]. Understanding whether changes in health-related QoL scores are clinically significant is crucial for evaluating interventions [155,156]. The minimal clinically important difference is the smallest score change perceived as beneficial and justifying a management adjustment [157]. To enhance QoL, improve dietary intake, and prevent weight loss in HNC patients, guidelines suggest weekly dietitian follow-ups during RT [139,158].

QoL evaluations of HNC patients are crucial because they enable healthcare staff to better inform and prepare patients for functional changes and symptoms during treatment. Additionally, understanding various health aspects aids in preventing and reducing functional impairments. Despite significant research on quality of life, function, and symptoms, some areas remain underexplored. A more quantitative assessment of nutritional status as a measure of QoL, as well as a more dynamic approach to evaluate QoL from a nutritional perspective, at different time points such as before, during, and after treatment, may enable proactive interventions before significant health deterioration occurs. By continuously analyzing data from wearables, electronic health records, and patient-reported outcomes, digital twins may provide personalized recommendations to optimize symptom management, nutritional intake, and physical activity, thereby enhancing overall well-being.

Furthermore, DT technology enables the simulation of treatment responses [159] and may aid oncologists and patients to evaluate different therapeutic strategies and their potential impact on fatigue, pain, mobility, and emotional health. HNC patients have a lower quality of life at diagnosis compared to the general population, which declines further during and shortly after treatment [160,161]. Women and patients over 65 years of age score lower than men and younger patients, respectively, in QoL assessments [162,163]. Factors such as tumor size, smoking, alcohol use, depression, social networks, personality, and marital status also impact quality of life in these patients [164,165,166,167,168,169].

Two parameters related to nutritional status may impact QoL in HNC patients: BMI and FFMI. BMI influences treatment outcomes [170], as obesity (BMI ≥ 30) is associated with reduced quality of life and physical activity, among other negative effects [171]. On the other hand, the FFMI serves as a predictor of overall survival and is linked to QoL [172,173]. Malnutrition thresholds are defined as FFMI <15 kg/m^2^ for women and <17 kg/m^2^ for men [121,174]. Additionally, a reduced phase angle, reflecting body cell integrity and mass, is a key marker of malnutrition and survival outcomes [175,176,177].

Integrating a broader range of nutritional status variables into comprehensive models may significantly enhance the predictive accuracy of a cancer patient’s nutritional status and QoL outcomes. Beyond metrics like BMI and FFMI, incorporating skeletal muscle mass, visceral fat levels, metabolic rate, and inflammatory markers may provide a broader view of a patient’s nutritional health. These variables, when combined with machine learning and artificial intelligence-driven simulations, might improve the predictive power of DT models in assessing malnutrition risk, cachexia progression, treatment tolerance, and long-term survivorship outcomes.

#### 4.5.3. Limitations of Current Data Landscape of QoL and Nutritional Status

Reporting is inconsistent across the included studies, with speech, swallowing, and social eating being some of the most frequently documented QoL related variables, with others such as financial difficulties and sexuality being the least documented. Furthermore, there are differences in the reporting metrics (i.e., median versus mean). A significant limitation of the heterogeneous data presented is the variability in study designs, methodologies, and reporting formats across the included studies.

This variability makes it challenging to generalize findings across the broader population of HNC patients. Apart from this, there is no uniform longitudinal reporting or no longitudinal reporting at all, as seen in cross-sectional studies. This could also be an issue in assessing QoL in the long term.

Patient-reported QoL parameters are subjective based on individual perception. This fact could cause concern regarding bias. Moreover, patient particularities like comorbid conditions or treatment-related aspects like tailored nutritional interventions or differing anti-cancer medications or RT regimens could further cause difficulties in evaluating QoL.

#### 4.5.4. Personalization of Interventions by Comprehensive Description of Disease Complexity

The previously mentioned limitations indicate the need for proper universally available tools to evaluate nutritional status and, most importantly, to support decision-making for screening, prevention measures, and nutritional interventions. Such tools should include risks models based on large databases, including a wide range of variables capable of satisfactorily and comprehensively describing the disease complexity.

Beyond patient (age, sex, and demographics) and disease (tumor type, localization, and staging) description, risk models should also include the risks and prognostics factors. For instance, common risk factors for HNSCC include smoking and heavy alcohol consumption. Patient-related prognostic factors for HNC include features such as smoking, alcohol use, performance status, and age, alongside TNM stage and HPV status, notably HPV-16 in the case of oropharyngeal cancers and EBV DNA in the case of nasopharyngeal cancers. HPV-related oropharyngeal cancers exhibit differences from those arising from traditional risk factors, typically affecting younger patients without tobacco or alcohol exposure. These cancers tend to have a milder disease course and fewer distant metastases. Interestingly, HPV-positive patients experience worse swallowing and sticky saliva scores [52], indicating the need for targeted supportive care.

Recent risk stratification models for HNSCC are based on genetics, epigenetics, and various OMICs (radiomics, metabolomics, and dosiomics) [178,179,180,181]; however, prediction models for treatment-related tissue toxicities are lacking and are limited due to being based solely on treatment planning dosimetry [182,183] or on radiomics [184,185].

Another example of needed variables is mental health, which is both a descriptor of QoL and a determinant of nutrition, being associated with appetite and nutrition-related behaviors. Reported increased Beck Depression Inventory (BDI) scores (in 83.5% of HNC patients) in recent nutrition-related QoL studies [17] highlight a decline in mental health, potentially related to treatment stress, disease progression, or other psychosocial factors. With most participants experiencing increased depressive symptoms, early intervention and mental health support could both improve QoL outcomes and ameliorate nutrition-related behaviors. Challenges in social interactions appear to increase slightly post-treatment (with no statistical significance), which could be related to physical or psychological changes caused by radiotherapy or its side effects [42]. Increased awareness may reflect patients’ adaptation or better understanding of their condition over time. The lack of statistical significance [42] suggests that this is not a universal trend and may vary widely among individuals.

Although ECOG is considered more of a predictor of post-therapeutic outcomes, its definition of patient autonomy makes it suitable as a descriptive variable of QoL. Regardless of the category of patients according to BMI, the ECOG score gradually worsens with the administration of RT [47], with some studies reporting that up to two-thirds of patients have worsened performance status while the rest maintain their performance status during RT [17]. The results suggest the need for interventions to mitigate factors causing decline, including worsening nutrition. Tracking ECOG changes over time can help adjust treatment plans and focus on QoL.

Implementing standardized protocols for the prevention and management of adverse effects, along with utilizing DT technology through the integration of patient health records and real-time data, has the potential to significantly enhance HNC patient outcomes and promote the adoption of more personalized treatment approaches. Additionally, the relationships between adverse effect management, various treatment regimens such as RT or CCRT, and risk factors—including age, smoking, alcohol consumption, and comorbidities like diabetes or conditions that elevate infection risk—demand more extensive research to better inform clinical practices and optimize patient care.

## 5. Conclusions

Although the problem of improving quality of care in NCH patients has been studied quite extensively in recent decades, there is unfortunately no uniform view on risk and prognostic models related to nutritional status and malnutrition as categories of QoL. In this review, we identified a number of clinical and demographic variables (age, gender, loss of pregnancy, BMI, education, tumor location, staging, etc.) that could be the basis of models capable of capturing the complexity of the disease and providing risk estimates and prognosis regarding the nutritional component of QoL in these patients. Although reported in numerous studies, they are not integrated into risk models even if they are simplified. Along with other adverse effects and the inherent inflammatory status of the disease, oral mucositis is an impediment that is difficult to circumvent in the decrease in the ability to eat and consequently to the appearance of malnutrition and decreased QoL. The QoL assessment is carried out through different questionnaires that have a descriptive, comprehensive character and that would be a starting point in the design of more advanced digital tools for data collection and other evolutionary models based on machine learning. Obtaining estimated risks, predictions, and, therefore, a basis for decision-making regarding personalized interventions could be achieved by integrating these models into a digital twin patient that contains, in addition to clinical data such as those listed above, known risk factors and other newly identified ones. Our review makes a fairly complete catalog of clinical, demographic, and therapeutic variables and applications of possible digital solutions to nutritional interventions (surgical enteral nutrition, or non-surgical counseling) and variables for evaluating their effectiveness. Also, this review suggests the need for large-scale studies on QoL in HNC patients, whose successful implementation seems to be conditioned by the existence of digital tools and rigorous methodologies.

## Figures and Tables

**Table 1:** The dimensions (N) of different patient cohorts from 10 different studies, as well as the distribution by sex and age groups of these cohorts (in absolute values and relative frequencies, i.e., percentages). When the distribution by age groups was not reported, we included the mean value for the entire cohort or the average values of the sub-cohorts (as is the case in [52]).

		**Kartin et al., 2014 [48]**			**Egestad and Nieder, 2015 [47]**		**Kelvianto et al., 2018 [18]**			**Atasoy et al. 2020 [49]**		**Fanetti et al., 2021 [29]**		
**N**		**50**			**60**		**61**			**29**		**179**		
Sex		N		%	N	%	N		%	N	%	N		%
	M	43		86	46	76.66	47		77.04	24	82.75	127		70.94
	F	7		14	14	23.33	14		22.95	5	17.24	52		29.05
Age		Age group	N	%	Mean		Age group	N	%	Mean		Age group	N	%
		<39	5	10	60		18–29	5	8.2	56.79		<55	64	35.8
		40–49	6	12			30–39	15	24.6			55–64	52	29.1
		50–59	18	36			40–49	13	21.3			>=65	63	35.2
		>60	21	42			>=50	28	45.9					
		**Milecki et al., 2021 [42]**				**De Oliveira Faria et al., 2022 [50]**		**Löser et al., 2022 [52]**		**Blake et al., 2023 [51]**		**Pytel et al., 2023 [17]**		
**N**		**92**				**80**		**58**		**110**		**85**		
Sex		N	%			N	%	N	%	N	%	N	%	
	M	74	80.43			65	81.25	41	70.68	93	84.54	55	64.70	
	F	18	19.56			15	18.75	17	29.31	17	15.45	30	35.29	
Age		Age group	N		%	N/A		Mean		Mean		Age group	N	%
		<60	57		62			Malnourished—68.6		60.6		18–35	2	2.4
		>60	35		38			well nourished—62.5				36–45	12	14.1
												46–55	12	14.1
												56–65	33	38.8
												>65	30	30.6

**Table 2:** Treatment modalities administered to patients included in various studies reporting adverse effects on head and neck cancer patients.

	Egestad and Nieder, 2015 [47]		Kelvianto et al., 2018 [18]		Atasoy et al., 2020 [49]		Fanetti et al., 2021 [29]		De Oliveira Faria et al., 2022 [50]		Löser et al., 2022 [52]		Blake et al., 2023 [51]	
N	60		61		29		179		80		58		110	
	N	%	N	%	N	%	N	%	N	%	N	%	N	%
Radiotherapy	12	20.00	16	26.22	0	0	0	0	37	46.25	0	0	9	8.18
Chemotherapy	21	35.00	0	0	6	20.68	141	78.77	0	0	0	0	0	0
Chemoradiation	0	0	45	73.77	25	86.20	38	21.22	43	53.75	58	100	101	91.81
Surgery	0	0	0	0	0	0	0	0	0	0	0	0	20	18.18

**Table 3:** Radiotherapy and chemotherapy doses administered to patients included in various studies reporting adverse effects on head and neck cancer patients.

	Egestad and Nieder, 2015 [47]	Kelvianto et al., 2018 [18]	Atasoy et al., 2020 [49]	Fanetti et al., 2021 [29]	Löser et al., 2022 [52]	Blake et al., 2023 [51]
Radiotherapy	60–70 Gy	Minimum 30–40 Gy	60–70 Gy	(*) 70.95 Gy to PTV macro 62.70 Gy to PTV HR 56.10 Gy to PTV LR	60–70.4 Gy	60–70 Gy
Chemotherapy	Not specified	Not specified	Weekly cisplatin-based treatment for most patients; no dose specified	(**) Cisplatin at 80–100 mg/m^2^ q3w or (***) induction with 3 cycles of cisplatin (75 mg/m^2^) and DTX (75 mg/m^2^), 5-FU (750 mg/m^2^/day) and then weekly cisplatin at 40 mg/m^2^ during RT	Cisplatin 100 mg/m^2^ q3w or weekly cisplatin 40 mg/m^2^ or 5-FU (600 mg/m^2^) and MMC (10 mg/m^2^)	Cisplatin at 100 mg/m^2^ q3w or weekly cisplatin at 40 mg/m^2^ or cetuximab at 400 mg/m^2^ IV loading dose 1 week before RT and then weekly 250 mg/m^2^ during RT

PTV macro—planning target volume of macroscopic disease; PTV HR—planning target volume high risk; PTV LR—planning target volume low risk; q3w—every three-week administration; DTX—docetaxel; 5-FU—5-fluorouracil; MMC—mitomycin C. (*) All doses administered in 33 fractions; (**) for T1–T3 and N0–N1-staged patients; (***) for T4 and/or N2– N3-staged patients. For Fanetti et al. (2021) [29], induction chemotherapy was administered as follows: cisplatin and DTX on day 1, 5-FU on days 1–5, repeated every 3 weeks. For Löser et al. (2022) [52], 5-FU was administered on days 1–5 and MMC on days 5–36.

**Table 4:** The correlation coefficients between total protein intake, the ratio of protein intake to weight, the quality of protein intake, the prognostic mutational index (PNI), and the QoL score-variables from the EORTC QLQ-C30 questionnaire [18]. Bold values of the correlation coefficients represent values for which *p* < 0.05.

	Correlation Coefficient (r)			
Score Variable	Total Prot. Intake	Prot. Intake/Weight	Quality of Prot. Intake	PNI
General condition	0.13	0.03	−0.03	0.1
Physical function	0.07	−0.08	0.1	**0.4**
Role function	−0.2	−0.06	0.03	0.14
Emotional function	0.2	0.15	−0.05	0.08
Cognitive function	0.07	0.01	−0.1	0.15
Social function	0.24	0.08	−0.04	0.25
Fatigue	**−0.28**	−0.2	0.2	0.02
Nausea and vomiting	**−0.26**	−0.2	0.02	−0.05
Pain	−0.09	−0.07	0.19	−0.15
Insomnia	0.03	0.12	0.2	−0.17
Dyspnea	−0.03	0.1	−0.12	**−0.26**
Loss of appetite	**−0.3**	**−0.3**	0.2	0.01
Constipation	−0.08	−0.04	0.07	−0.14
Diarrhea	−0.09	0.003	−0.03	0.2
Financial difficulties				

**Table 5:** The median of the EORTC QLQ-C30 questionnaire baseline score values [18] and the mean values of the score variables in different cohorts defined by body mass index (BMI) values (≥25 vs. <25) at the beginning and end of treatment [47], at the time of radiotherapy (RT) administration completion [47], age groups, and adherence to nutritional interventions [50] from the questionnaire. We marked in bold the mean values for which the mean comparison test across sub-cohorts revealed statistically significant differences (*p* ≤ 0.05).

Study	Kelvianto et al., 2018 [18]	Egestad and Nieder, 2015 [47]				Milecki et al., 2021 [42]				De Oliveira Faria et al., 2022 [50]	
	Median	Means (time point and BMI)				Means (follow-up)		Means (age groups)		Means (by adherence to intervention)	
Score Variable	Baseline	Treatment start, BMI ≥ 25	Treatment start, BMI < 25	Treatment end, BMI ≥ 25	Treatment end, BMI < 25	Before RT	1 year after RT	≤60 yr	>60 yr	Adherent	Non-adherent
General condition	58.3	68.6	65.6	48.3	47.8	61.14	57.88			76.7	73.2
Physical function	66.7	83.1	84.5	69.1	65.3	**79.93**	**68.72**			72.9	73.1
Role function	66.7	71.1	76.4	44.8	43.6	**83.15**	**75.72**			53.1	50.9
Emotional function	75	81.5	87.2	75.0	77.2			**67.98**	**73.40**	75.6	70.7
Cognitive function	100	89.2	86.1	78.3	67.8	**84.06**	**73.73**			88.8	92.8
Social function	66.7	71.1	73.6	60.1	60.9	79.89	75.18			82.9	86.0
Fatigue	16.7	32.3	31.9	53.7	62.3	**31.28**	**39.85**			20.4	18.6
Nausea and vomiting	16.7	13.0	17.4	33.3	25.4	7.07	9.78			13.9	22.5
Pain	16.7	15.2	18.1	44.4	53.6	22.28	28.44	**24.54**	**17.52**	14.3	20.8
Insomnia	0	26.5	33.3	25.6	34.8	34.42	39.86			27.9	33.3
Dyspnea	0	**22.5**	16.7	22.2	30.4					18.6	10.8
Loss of appetite	33.3	17.6	22.2	57.8	66.7	**20.65**	**27.17**			27.9	27.8
Constipation	0	17.2	20.8	46.7	47.8	**15.58**	**21.38**			27.9	19.8
Diarrhea	0	14.1	13.9	17.8	13.6	7.25	6.88			5.4	5.4
Financial difficulties		13.7	18.1	15.6	30.3	33.70	38.04	**39.89**	**30.34**	34.9	36.9

BMI—Body Mass Index; RT—Radiotherapy.

**Table 6:** Associations of different score variables from EORTC QLQ H&N35 questionnaire through correlation coefficients with variables defined in relation to protein intake and PNI and through regression coefficients with values of scores form other questionnaires: Malnutrition Universal Screening Tool (MUST); Nutritional Risk Screening 2002 (NRS-2002); Human Papillomavirus (HPV); and Union Internationale Contre le Cancer (UICC). Statistically significant values (*p* ≤ 0.05) of correlation and regression coefficients are bolded.

	Correlation Coefficient (r) [18]				Regression Coefficient (β) [52]			
Score Variable	Total Prot. Intake	Prot. Intake/Weight	Quality of Prot. Intake	PNI	MUST	NRS-2002	HPV Status	UICC
Mouth pain	**−0.32**	−0.17	0.19	−0.7				
Speech	−0.03	0.17	0.2	−0.05				
Swallowing	**−0.37**	−0.15	0.04	−0.2		**16.8**	**24.9**	
Senses (taste and smell)	−0.15	−0.64	0.05	−0.05				
Teeth	0.013	0.17	0.07	−0.5				
Opening mouth	−0.13	0.11	0.04	**−0.32**				
Dry mouth	**−0.41**	**−0.3**	0.02	−0.2				
Sticky saliva	**−0.32**	−0.2	0.04	−0.2		**38.1**	**36.3**	
Coughing	0.17	0.13	0.11	−0.11				
Feeling ill	0.04	0.17	0.21	0.4				
Weight loss						**51**		**39.1**
Weight gain								
Pain medication								
Feeding tube					**35.6**			
Food supplements								
Appetite loss								
Social eating	**−0.29**	−0.26	0.14	0.08				
Social contacts								
Awareness of disease								
Sexuality								

**Table 7:** The mean and median values of the score variables from the EORTC QLQ H&N35 questionnaire for defined sub-cohorts in relation to the time point [17,18,42,47,49], nutritional status [52], or adherence to nutritional intervention [50]. Values with statistically significant differences (*p* ≤ 0.05) are bolded.

	Kelvianto et al., 2018 [18]	Atasoy et al., 2020 [49]		Egestad and Nieder, 2015 [47]				Milecki et al., 2021 [42]		Löser et al., 2022 [52]		De Oliveira Faria et al., 2022 [50]		Pytel et al., 2023 [17]	
Parameter	Median	Median		Means (time point and BMI)				Means (follow-up)		Medians (nutrition status)		Means (by adherence to intervention)		Mean (time point)	
	Baseline	RT start	RT end	Treatment start, BMI ≥ 25	Treatment start, BMI < 25	Treatment end, BMI ≥ 25	Treatment end, BMI < 25	Before RT	1 year after RT	Mal-nourished	Well nourished	Adherent	Non-adherent	Before RT	After RT
Mouth pain	25	**8.33**	**33.3**	17.2	18.0	49.4	48.2	22.16	28.11			13.9	16.9		
Speech	44.4	**0**	**77.8**	13.1	23.1	39.8	47.0	35.63	33.45	**33.3**	**0**	38.2	37.2	**31.9**	**56.9**
Swallowing	41.6	**8.33**	**33.3**	6.9	11.6	45.7	45.3	19.93	23.64	**58.3**	**12.5**	**43.0**	**25.4**	**31.4**	**66.4**
Senses (taste and smell)	50	**100**	**0**	14.2	26.7	50.6	55.8	**19.3**	**35.69**			24.0	29.3	**26.3**	**71.2**
Teeth	0			4.9	10.1	14.9	10.6					56.1	47.7	50.0	50.9
Opening mouth	33.3			17.6	9.3	40.2	39.1	**22.10**	**30.07**			27.9	24.3	50.0	66.5
Dry mouth	66.7			29.4	41.3	**64.4**	62.3	**26.81**	**51.09**			34.1	27.9	0	75.9
Sticky saliva	66.7			27.3	34.7	71.4	68.1	**28.62**	**42.75**	**66.7**	**0**	53.5	50.4	25.0	76.2
Coughing	33.3			20.6	21.3	35.6	43.5					**41.1**	**16.2**	**33.8**	**64.1**
Feeling ill	33.3			16.7	17.8	43.7	45.5					14.7	16.2	**33.2**	**59.4**
Weight loss				14.7	29.2	62.1	63.6	**60.87**	**78.26**	**100**	**0**			**38.8**	**29.7**
Weight gain				30.3	43.5	7.4	4.8	57.61	60.87	**0**	**0**			48.5	48.5
Pain medication				44.1	40.0	86.7	95.5	**52.75**	**6.96**	**0**	**0**			**43.8**	**25.9**
Feeding tube				**0**	12.5	30.0	54.5			**0**	**0**			46.8	46.8
Food supplements				**3.0**	36.0	46.7	68.2	72.83	76.09						
Appetite loss										**16.7**	**66.7**				
Social eating		**0**	**16.6**	9.8	18.8	48.3	49.9	20.02	25.36	**41.7**	**8.3**	46.9	44.6	**30.9**	**66.9**
Social contact				4.2	6.1	23.0	28.9	17.46	21.96			15.2	14.6	**28.5**	**45.8**
Awareness of disease								25.00	28.98						
Sexuality				30.1	34.8	53.2	41.6	35.51	39.13			43.6	44.6	34.0	45.5

BMI—body mass index; RT—radiotherapy.

## Abbreviations

AE	adverse effects
BMI	body mass index
CCRT	chemoradiation therapy
DT	digital twin
EBV	Epstein–Barr virus
EORTC	European Organization for Research and Treatment of Cancer
ESPEN	European Society for Clinical Nutrition and Metabolism
FFM	fat-free mass
FFMI	fat-free mass index
HNCs	head and neck cancers
HNSCC	head and neck squamous cell carcinomas
HPV	Human Papillomavirus
IMRT	intensity-modulated radiotherapy
MUST	Malnutrition Universal Screening Tool
NRS-2002	Nutritional Risk Screening 2002
QoL	quality of life
RT	radiotherapy
UICC	Union Internationale Contre le Cancer
